# Comment on: “GLM7 – A Novel Composite Glycolipid Index Derived from Routine Health Indicators for Enhanced Diagnosis and Prediction of Multimorbidity”

**DOI:** 10.1002/advs.74610

**Published:** 2026-03-02

**Authors:** Yiquan Wang, Tin‐Yeh Huang

**Affiliations:** ^1^ College of Mathematics and System Science Xinjiang University Urumqi Xinjiang China; ^2^ Department of Industrial and Systems Engineering The Hong Kong Polytechnic University Hong Kong China

## Abstract

The recent study by Wang et al. introduces GLM7, a novel composite index integrating routine health indicators to enhance the diagnosis and prediction of multimorbidity. The study provides compelling evidence for the utility of cost‐effective biomarkers in large‐scale health screening. In this comment, we engage in a constructive discussion regarding the methodological interpretation of the index. Specifically, we explore the implications of incorporating diagnostic criteria (such as fasting blood glucose) into predictive models for diabetes, the mathematical nuance required when comparing Odds Ratios (ORs) between logarithmic indices and linear variables, and the structural contribution of age within the composite formula. We believe that addressing these aspects could further solidify the clinical applicability and interpretability of GLM7.

We read with great interest the article by Wang et al. titled “GLM7 – A Novel Composite Glycolipid Index Derived from Routine Health Indicators for Enhanced Diagnosis and Prediction of Multimorbidity” [1]. We congratulate the authors on this extensive study utilizing the NHANES and CHARLS cohorts to validate a unified health index. The effort to synthesize routine indicators into a single, interpretable metric aligns perfectly with the goals of precision medicine and accessible healthcare. While the findings are robust, we would like to respectfully offer a discussion on three methodological aspects to further clarify the physiological and statistical interpretation of the GLM7 index.

## 1 Distinction Between Diagnosis and Prediction in Diabetes Models

The study reports an impressive Area Under the Curve (AUC) for Diabetes Mellitus (DM) prediction, reaching 0.98 in the training set. As defined in the study, the GLM7 index is calculated as:

(1) $$\text{GLM7}=\log_{10}\left(\frac{\text{Age}\times \text{BMI}\times \text{FBG}\times \text{Insulin}\times \text{TG}\times \text{LDL-c}}{\text{HDL-c}}\right)$$

We noticed that Fasting Blood Glucose (FBG) is a direct multiplier in the numerator. Since elevated FBG (≥126 mg dL^−1^) is a primary diagnostic criterion for diabetes according to standard guidelines [2], the inclusion of FBG in the GLM7 formula creates a strong intrinsic correlation with the disease outcome. In clinical prediction modeling, incorporating predictors that define the outcome is often cautioned against as it constitutes a form of data leakage or circularity, which may inflate performance metrics [3, 4].

Therefore, it might be beneficial to distinguish between the index's diagnostic capability (detecting existing diabetes) and its prognostic value (predicting future risk). We wonder if the authors have considered assessing the predictive performance of a modified version of the model—one that minimizes the weight of direct glucose parameters—to evaluate the index's independent contribution to risk stratification beyond standard glycemic definitions.

## 2 Standardization of Odds Ratios Across Scales

The study highlights that the Odds Ratio (OR) for GLM7 (ranging from 9.98 to 12.19) is significantly higher than that of traditional risk factors such as Age (OR ≈ 1.09). We suggest that this comparison warrants a careful look at the measurement scales.

The OR for Age typically reflects the risk increase associated with a 1‐year increment. However, GLM7 is a base‐10 logarithmic index. Mathematically, a 1‐unit increase in GLM7 corresponds to a tenfold increase in the underlying product of the metabolic components:

$$\Delta \text{GLM7}=1\Rightarrow \frac{{\text{Product}}_{\text{new}}}{{\text{Product}}_{\text{old}}}=10$$

A tenfold increase in the combined metabolic parameters represents a drastic physiological change, whereas a 1‐year increase in age is a relatively small increment. Consequently, comparing the raw OR of a log‐transformed variable against a linear variable might inadvertently inflate the perceived relative strength of the former.

To provide a more standardized comparison, we respectfully suggest presenting ORs per Standard Deviation (SD) increase or per Interquartile Range (IQR) for all continuous variables [5]. This adjustment would place GLM7 and Age on a comparable statistical footing, allowing readers to gauge their relative predictive strengths more accurately.

## 3 Structural Contribution of Age in Non‐Metabolic Diseases

The GLM7 index demonstrates versatility by predicting non‐metabolic conditions, such as Cancer. We note that Age is included as a multiplier in the numerator of the formula (Equation 1). Since age is universally recognized as the dominant risk factor for cancer and multimorbidity [6], there is a possibility that the index's performance in these specific categories is largely driven by the age component.

While the study performs subgroup analyses stratified by age, the structural dependence of the formula on age remains. It would be scientifically valuable to understand the added value of the glycolipid components specifically. A sensitivity analysis comparing the AUC of GLM7 against Age alone (or a model containing only Age and BMI) would help illuminate the specific contribution of the metabolic interaction to cancer prediction. If GLM7 significantly outperforms Age alone, it would strongly support the hypothesis that dysregulated glycolipid metabolism is a key driver of these comorbidities independent of aging.

## 4 Conclusion

The GLM7 index represents a promising step toward integrating routine health data for comprehensive risk assessment. Our comments aim to refine the interpretation of its components and statistical metrics. We look forward to the authors' insights, which will undoubtedly enhance the robustness and clinical translation of this novel index.

## Author Contributions

**Yiquan Wang**: Conceptualization, writing – original draft, writing – review & editing. **Tin‐Yeh Huang**: writing – original draft, writing – review & editing. All authors have read and agreed to the published version of the manuscript.

## Conflicts of Interest

The authors declare no conflicts of interest.

## Data Availability

Data sharing not applicable to this article as no datasets were generated or analyzed during the current study.
